# Albumin versus crystalloid solutions in patients with the acute respiratory distress syndrome: a systematic review and meta-analysis

**DOI:** 10.1186/cc13187

**Published:** 2014-01-09

**Authors:** Christopher Uhlig, Pedro L Silva, Stefanie Deckert, Jochen Schmitt, Marcelo Gama de Abreu

**Affiliations:** 1 Pulmonary Engineering Group, Department of Anesthesiology and Intensive Care Therapy, University Hospital Dresden, Technische Universität Dresden, Dresden, Germany; 2 Laboratory of Pulmonary Investigation, Carlos Chagas Filho Biophysics Institute, Federal University of Rio de Janeiro, Rio de Janeiro, Brazil; 3 Center for Evidence-based Healthcare, University Hospital Dresden, Technische Universität Dresden, Dresden, Germany

## Abstract

**Introduction:**

In patients with acute respiratory distress syndrome (ARDS) fluid therapy might be necessary. The aim of this systematic review and meta-analysis is to determine the effects of colloid therapy compared to crystalloids on mortality and oxygenation in adults with ARDS.

**Methods:**

Randomized controlled trials (RCTs) were identified through a systematic literature search of MEDLINE, EMBASE, CENTRAL and LILACS. Articles published up to 15^th^ February 2013 were independently screened, abstracted, and assessed (Cochrane Risk of Bias Tool) to provide evidence-based therapy recommendations. RCTs were eligible if they compared colloid versus crystalloid therapy on lung function, inflammation, damage or mortality in adults with ARDS. Primary outcome parameters were respiratory mechanics, gas exchange lung inflammation and damage as well as hospital mortality. Kidney function, need for renal replacement therapy, hemodynamic stabilization and intensive care unit (ICU) length of stay served as secondary outcomes.

**Results:**

A total of 3 RCTs out of 4130 potential trials found in the databases were selected for qualitative and quantitative analysis totaling 206 patients who received either albumin or saline. Overall risk of bias was unclear to high in the identified trials. Calculated pooled risk of death was not statistically significant (albumin 34 of 100 (34.0%) versus 40 of 104 (38.5%), relative risk (RR) = 0.89, 95% confidence interval (CI) 0.62 to 1.28, *P* = 0.539). Weighted mean difference (WMD) in PaO_2_/FiO_2_ (mmHg) improved in the first 48 hours (WMD = 62, 95% CI 47 to 77, *P* <0.001, *I*^*2*^ = 0%) after therapy start and remained stable after 7 days (WMD = 20, 95% CI 4 to 36, *P* = 0.017, *I*^*2*^ = 0%).

**Conclusions:**

There is a high need for RCTs investigating the effects of colloids in ARDS patients. Based on the findings of this review, colloid therapy with albumin improved oxygenation but did not affect mortality.

## Introduction

In patients suffering from acute respiratory distress syndrome (ARDS), the alveolar-capillary barrier permeability is increased due to inflammation, resulting in extravasation of protein-enriched fluid into the alveoli [1]. In turn, the presence of pulmonary exudate in the alveoli, as well as the inactivation of lung surfactant can result in life-threatening hypoxemia, impaired CO_2_ elimination, and decreased lung compliance [1]. Thus, mechanical ventilation is often required in such patients in order to improve oxygenation and alleviate the work of breathing. The use of low tidal volumes and moderate-to-high levels of positive end-expiratory pressure (PEEP) can reduce mortality in severe ARDS patients [2]. As it is a multifactorial syndrome, patients with ARDS face reduction of intravascular volume during the course of disease. In order to counteract these episodes, fluid therapy needs to be instituted promptly.

Experimental and clinical data demonstrate beneficial effects on the lungs of colloids compared to crystalloids, including reduced alveolar-capillary permeability [3], less histological damage [4], decreased inflammatory cell infiltration [5] and faster hemodynamic stabilization [6]. On the other hand, tissue edema might be increased due to extravasation of colloid molecules in the presence of high capillary leakage [7]. Furthermore, damage due to the use of synthetic colloids, namely hydroxylethlyl starch, and also gelatin are associated with a higher risk of death and kidney injury in septic patients [8-10]. Due to those adverse effects, the interest in intravascular volume expansion using human albumin is increasing. However, a recent meta-analysis on the use of colloids in general critically ill populations found no difference in mortality among the different colloids compared to crystalloids [11].

In view of those controversial findings the use of colloids in critically ill patients has been intensively debated, but a consensus has not been achieved. In particular, the population of ARDS patients remains unaddressed [12]. Therefore, we performed a systematic review and meta-analysis to evaluate the efficacy of colloid compared to crystalloid therapy on mortality and oxygenation in adults with ARDS.

## Methods

### Study type and registration

We conducted a systematic review of randomized controlled trials (RCTs) in accordance with a previously registered protocol (PROSPERO, registration number CRD42012003162). The presented review was performed according to the preferred reporting items for systematic reviews and meta-analyses (PRISMA) statement [13].

### Identification of relevant studies

Four databases (EMBASE, MEDLINE, The Cochrane Central Register of Controlled Trials (CENTRAL) and LILACS) were systematically searched for relevant trials published from inception until 15 February 2013, without language restriction. Personnel files and reference lists of relevant review articles for additional trials were also reviewed. Detailed search strings are listed in Additional file 1.

### Eligibility criteria

Inclusion criteria with respect to the patient, population or problem, intervention, comparison, outcomes, and setting (PICOS) criteria [14] were as follows: 1) population: clinical diagnosis of acute lung injury (ALI) or ARDS as defined by the American-European consensus conference in 1994 [15], or the Berlin definition 2012 [16], or a ratio of arterial partial pressure of oxygen/fraction of inspired oxygen (PaO_2_/FiO_2_) lower than or equal to 300 during invasive mechanical ventilation, or some indication that the majority of patients would meet this criterion. Patients also had to be older than 16 years; 2) intervention: patients submitted to or requiring fluid therapy (main intervention or co-intervention); 3) comparison: colloids, independently of molecular weight compared to crystalloids must represent one of the fluid therapies; 4) outcome: primary outcome parameters were respiratory mechanics (compliance, plateau pressure) or gas exchange (arterial carbon dioxide partial pressure (PaCO_2_), PaO_2_/FiO_2_) or parameters of lung inflammation (bronchoalveolar lavage fluid neutrophils or IL-8 levels) and damage, hospital mortality; secondary outcome parameters: kidney function (creatinine, neutrophil gelatinase-associated lipocalin (NGAL) or need for renal replacement therapy (intermittent or continuous hemodialysis, hemofiltration or hemodiafiltration), hemodynamic stabilization (time and amount of fluid), ICU length of stay; and 5) design: RCT. Trials were excluded if they were uncontrolled, pseudo-randomized, published only as an abstract, or if all intervention groups received colloid therapy.

### Trial selection and data abstraction

The articles for this review were selected by examining titles, abstracts, and the full text if a potentially relevant trial was identified. We translated non-English reports into English, as necessary. Two reviewers (CU, PLS), independently and in duplicate, abstracted the data on the a priori-defined inclusion criteria (population, intervention, comparison, clinical outcomes and design). Trial data presented in figures only were extracted using Engauge Digitizer (Version 5.1., http://digitizer.sourceforge.net).

### Risk of bias assessment and strength of evidence

In duplicate and independently, two reviewers assessed trial methodological quality using the risk-of-bias tool recommended by the Cochrane Collaboration [17]. For each trial, the risk of bias was reported as low risk, unclear risk, or high risk in the following domains: random sequence generation, allocation concealment, blinding of participants and personnel, blinding of outcome assessment, incomplete outcome data, selective reporting, and other bias [17]. For each outcome, we independently rated the overall quality of evidence (confidence in effect estimates) in duplicate using the GRADE approach in which trials begin as high-quality evidence, but may be rated down by one or more of five categories of limitations: risk of bias, inconsistency, indirectness, imprecision, and reporting bias [18]. Finally, the overall risk of bias for an individual trial was categorized as low (if the risk of bias was low in all domains), unclear (if the risk of bias was unclear in at least one domain, with no high risk of bias domains), or high (if the risk of bias was high in one or more domains). Disagreement was resolved by discussion and consensus. Attempts were made to contact the authors and request for any necessary information not contained in the publications.

### Data synthesis

All analyses were performed using STATA (Version MP 11, Stata Corp LP, Lake Drive, TX, USA). To calculate the pooled risk ratio (RR) and 95% CIs of binary outcomes (mortality) trial data were combined using the Mantel-Haenszel estimator with estimation of variances according to the DerSimonian and Laird random-effect model [19]. For continuous outcomes, the pooled weighted mean difference (WMD) in the gas exchange (PaO_2_/FiO_2_) was calculated weighting the effect in respect to sample size. Statistical heterogeneity was assessed by the *I*^*2*^ statistic. Substantial heterogeneity was predefined as *I*^*2*^ >50%.

## Results

### Trial identification

**Figure 1:**
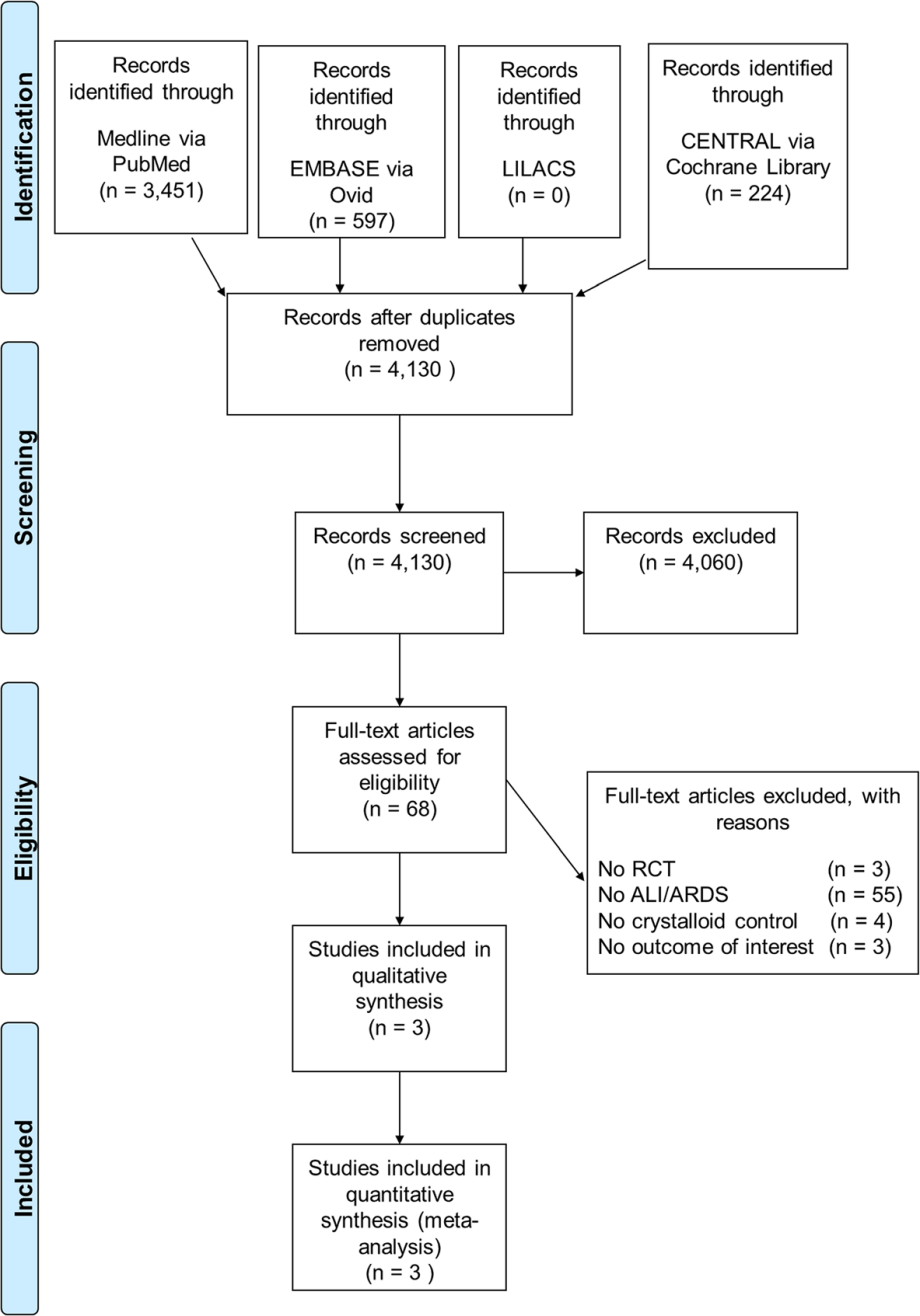
**Data extraction flow chart.** RCT, randomized controlled trial; ALI, acute lung injury; ARDS, acute respiratory distress syndrome.

The search yielded 4,130 publications. The flowchart of the articles is depicted in Figure 1. One report was translated from Mandarin [20] and one from German [21] into English to access eligibility. Of 68 potentially eligible studies, three were excluded because they were not RCTs, 55 studies did not match the ALI or ARDS criteria, 4 trials were excluded due to fluids comparison [20,22-24], and 3 studies did not report the outcome investigated by this review [25-27]. Detailed information on the excluded articles is listed in the Additional file 2. Finally, two trials and one subgroup from a large RCT were included in this review, and their data were analyzed.

### Trial characteristics

Characteristics of the three trials (the two trials and one subgroup from a large RCT that were included in this review) are shown in Table 1. Two trials were published by the same group using 25% albumin as colloid therapy and basic diuretic therapy with furosemide compared to saline in patients with ALI [28,29]. The study of saline versus albumin fluid evaluation (SAFE trial) used 4% albumin compared to saline [30].

**Table 1:** Trial characteristics

**Trial**	**Population**		**Intervention**		**Outcome**	
**(sample size)**	**Inclusion criteria**	**Exclusion criteria**	**Treatment**	**Control**	**Primary**	**Secondary**
Martin [29] (n = 37)	1) The American-European Consensus Conference definition for ALI [14]; 2) serum total protein level of 5.0 g/dL; 3) ongoing nutritional support; 4) mechanical ventilation >48 h. Number of recruiting centers: 2	1) Hemodynamic instability; 2) renal disease; 3) hepatic failure; 4) allergies to albumin or furosemide; 5) age <18 or >80 years; 6) pregnancy; 7) serum sodium; >150 meq/L or potassium <2.5 meq/L	Albumin 25%, 25 g every 8 h (100 ml) for 5 days + furosemide continuous infusion titration total albumin dosage: 400 g (1,600 ml)	Saline 100 ml every 8 hr for 5 days + 0.9% saline continuous infusion	Change in body weight	Oxygenation; 30-day mortality; net fluid balance
Martin [28] (n = 40)	1) The American-European Consensus Conference definition for ALI [14]; 2) serum total protein level of 6.0 g/Dl; 3) ongoing nutritional support; 4) mechanical ventilation >24 h; 3) ongoing nutritional support; 4) mechanical ventilation >24 h. Number of recruiting centers: 4	1) Hemodynamic instability; 2) renal disease; 3) clinically documented cirrhosis; 4) allergies to albumin or furosemide; 5) age <18 or >80 years; 6) pregnancy; 7) serum sodium >155 meq/L or potassium of <2.5 meq/L	Albumin 25%, 25 g every 8 h for 3 days; furosemide continuous infusion titration total albumin dosage: 250 g (1,000 ml)	Saline 0.9% (100 ml) every 8 h for 3 days furosemide continuous infusion titration (1 mg/ml)	Change in oxygenation after 24 h	Net fluid balance; 30-day mortality; serum albumin; serum creatinine
SAFE [30] ARDS subgroup (n = 127)	1) Need for additional fluid resuscitation additional to intravenous fluid that was required for nutrition or to replace ongoing insensible losses, urinary losses, ongoing losses from other sites; 2) 4% human albumin solution and 0.9% sodium chloride were equally appropriate for the patient judged by treating physician; 3) requirement for fluid resuscitation must have been supported by at least one of the following clinical signs: a. HR >90 bpm; b. SBP <100 mmHg or MAP <75 mmHg or a 40 mmHg decrease in SBP or MAP from the baseline recording, or requirement for inotropes or vasopressors; c. CVP <10 mmHg; d. PCWP <12 mmHg; e. respiratory variation in systolic or mean arterial blood pressure >5 mmHg; f. capillary refill time >1 s; g. UOP <0.5 mL/kg for 1 h. Number of recruiting centers: 16	1) Adverse reaction to albumin; 2) religious objection to the administration of human blood products; 3) plasmapheresis during the ICU admission; 4) cardiac surgical patients; 5) patients with burns; 6) liver transplantation; 7) age <18 years; 8) brain dead; 9) low expectation of survival <24 h, not-to-be-resuscitated patients; 10) previous enrollment in the SAFE study; 11) previously received fluid resuscitation during current ICU or hospital admission; 12) previously received fluid resuscitation from transferring non-study ICU	Albumin 4% for all fluid resuscitation until ICU discharge, or death, or day 28; adaptive regime according to clinical status; total albumin dosage: not reported	Saline 0.9% for all fluid resuscitation during ICU discharge or death or until day 28; adaptive regime according to clinical status	28-day mortality	None reported for ARDS subgroup

ARDS, acute respiratory distress syndrome; ALI, acute lung injury; CVP, central venous pressure; HR, heart rate; MAP, mean arterial pressure; PCWP, pulmonary capillary wedge pressure; SAFE: saline versus albumin fluid evaluation; SBP, systolic blood pressure; UOP, urine output.

### Risk of bias

The Cochrane risk of bias tool is shown in Table 2, whereby risk of bias was assessed to be high, unclear or low. The overall risk of bias was unclear-to-high in the analyzed trials.

**Table 2:** Risk of bias assessment

**Trial**	**Sequence generation**	**Allocation concealment**	**Blinding of participants, personnel and outcome assessors**	**Incomplete outcome data**	**Selective outcome reporting**	**Other bias**	**Overall risk of bias**
Martin [29]	Low	Low	Low	Low	Unclear	High*	High*
Martin [28]	Low	Low	Low	Low	Unclear	Low	Unclear
SAFE [30]	Unclear	Low	Low	Low	Unclear	Low	Unclear

Sequence generation: Martin [28,29] via computer generated list (four-subject-block randomization), SAFE [30]: unclear risk of bias because of minimization strategy; allocation concealment: all trials no evidence for inadequate concealment of allocation prior to assignment; blinding: all trials had double blind design with camouflage of study drugs and infusion systems and blinding of assessors and patients; incomplete outcome data: all trials low risk for attrition bias for mortality and oxygenation; selective outcome reporting: all trials: unclear risk of bias due to no statement regarding this item in text or supplement; other bias: Martin [29]: *high risk of bias due to concomitant furosemide treatment, in resulting violations of study protocol due to furosemide side effects, but albumin therapy was continued. Martin [28] and SAFE trial: no evidence of other sources of bias.

### Mortality

**Figure 2:**
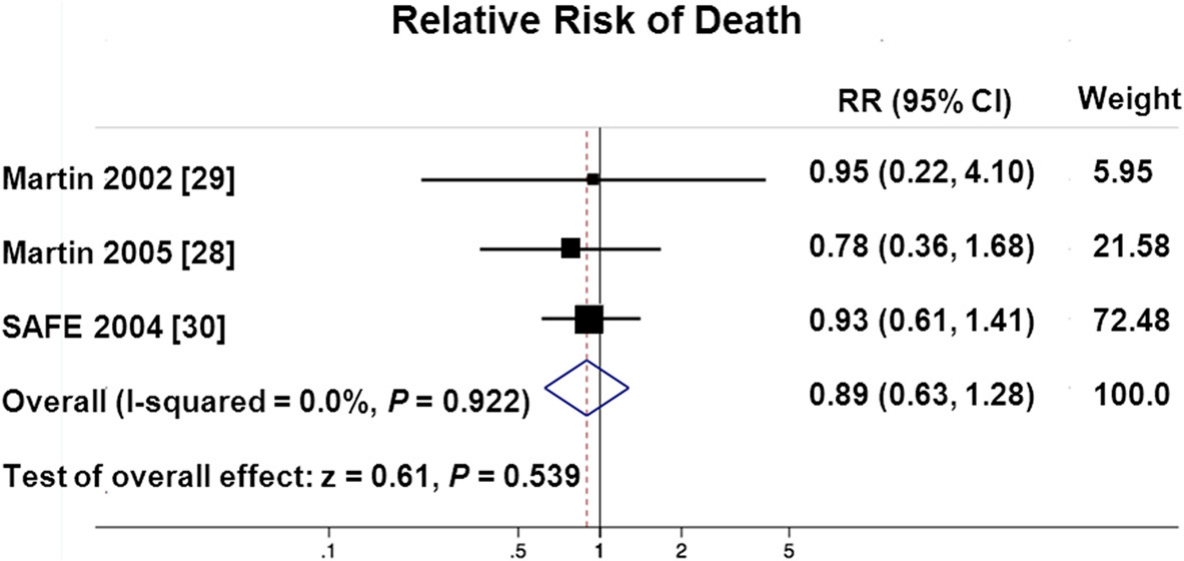
**Forest plot of pooled relative risk of death.** RR, relative risk; SAFE, saline versus albumin fluid evaluation trial.

Two trials [28,29] reported 30-day mortality and the 28-day mortality was reported for the subgroup of ARDS patients in the SAFE study (Figure 2). Albumin therapy did not significantly influence either 30-day mortality alone (albumin, 10 patients out of 39 (25.6%) versus control 12 patients out of 38 (31.6%), RR = 0.81, 95% CI 0.41, 1.60, *P* = 0.548), or combined pooled mortality including the SAFE trial (albumin, 34 patients out of 100 (34.0%) versus 40 out of 104 (38.5%), RR = 0.89, 95% CI 0.63, 1.28, *P* = 0.539).

### Oxygenation

**Figure 3:**
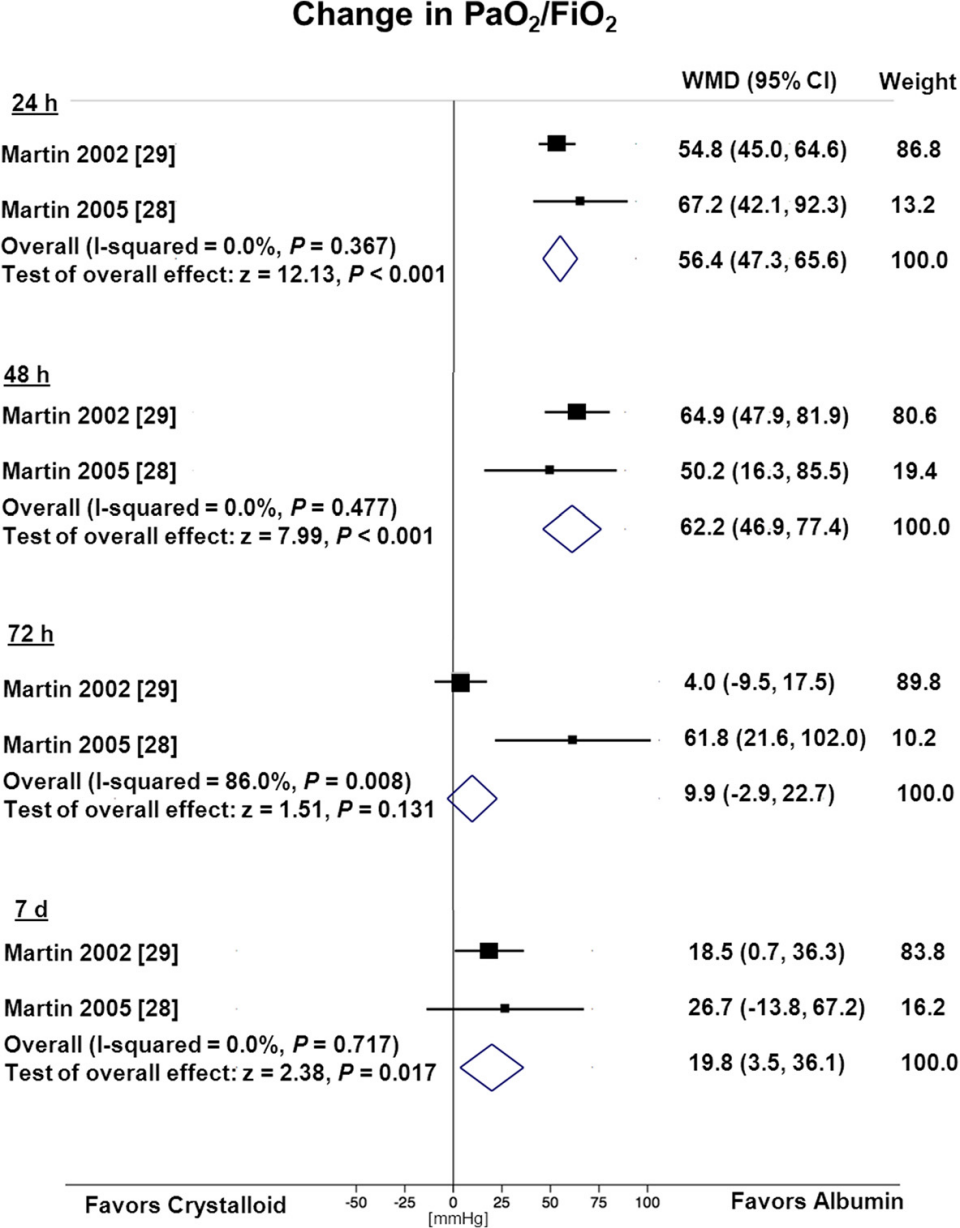
**Change in PaO**_**2**_**/FiO**_**2**_**.** PaO_2_/FiO_2_, ratio of arterial partial pressure of oxygen/fraction of inspired oxygen; WMD, weighted mean difference.

Two trials [28,29] reported change in oxygenation for ARDS patients (Figure 3). The WMD in change in PaO_2_/FiO_2_ significantly increased after albumin therapy in the first 24 h (WMD = 56 mmHg, 95% CI 47, 66, *P* <0.001, *I*^*2*^ = 0%) and 48 h (WMD = 62 mmHg, 95% CI 47, 77, *P* <0.001, *I*^*2*^ = 0%) as well as after 7 days (WMD = 20 mmHg, 95% CI 4, 36, *P* = 0.017, *I*^*2*^ = 0%). However, after 72 h, oxygenation did not differ between patients receiving albumin compared to crystalloids (WMD = 10 mmHg, 95% CI −3 - 23, p = 0.131, *I*^*2*^ = 86%).

## Discussion

In the present systematic review and meta-analysis, we identified three studies that compared albumin with crystalloid solutions for intravascular volume expansion in patients with ARDS. Meta-analyzing those data, we found that albumin improved oxygenation compared to crystalloids during early treatment, without affecting mortality.

A better understanding of the physiology of the endothelium, its changes during lung injury and the proposed new model of fluid filtration along the capillary may contribute to improve the treatment of patients with ARDS [31]. It is well known that the integrity of the capillary endothelium depends mainly on adherens junctions, whereby the vascular endothelial (VE)-cadherin represents one of the most important adheren junctions forming cadherins for endothelial cells. Recent studies in lung specimens of patients with ARDS have shown a reduced expression of VE-cadherin [32]. *In vitro* studies suggest that colloid expanders stabilize microvessels via physical mechanisms that enhance VE-cadherin localization at junctions and thereby limit vascular leakiness [33].

The findings of the present systematic review and meta-analysis are likely explained by the colloid osmotic pressure in the capillary. When this is elevated, as is theoretically the case with albumin solutions, alveolar-capillary leakage may be reduced. Clinical evidence so far suggests improved PaO_2_/FiO_2_ in the first two days and seven days after therapy start, supporting the hypothesis of reduced alveolar-capillary leakage. Nevertheless, the administration of albumin solutions failed to improve oxygenation 72 h after the primary insult. There are different non-mutually exclusive explanations for this discrepancy. First, differences in the duration of albumin therapy (three days versus five days), as suggested by the heterogeneity of data (*I*^2^ = 86%), may explain these findings. Second, it is possible that the alveolar-capillary leakage varied over time, with more pronounced changes in the first 48 h. Third, fluids may have also accumulated in the lungs during the transition from the early to the late phase of ARDS, for example, due to breakdown of the extra-cellular matrix [34], slightly deteriorating the oxygenation capability over time. Fourth, it is also conceivable that accumulation of fluids in the interstitium may have changed the mechanical properties of the lung tissue, increased transpulmonary pressure, and further deteriorated the lung structure.

This systematic review and meta-analysis has several limitations that need to be acknowledged. First, only three trials were included in the qualitative and quantitative analysis summarizing 204 patients, whereby the ARDS patients from the SAFE study accounted for 62% of the data, and only mortality was reported for this subgroup. A trial on the effects of colloids in critically ill patient populations, especially those with severe sepsis [9,35], likely included ARDS patients as well, but subgroup analyses have not been published. Second, only studies that used albumin solutions have been investigated. Thus, no conclusions about other colloids, or in general, can be drawn from this systematic review. Furthermore, in both trials published by Martin and colleagues [28,29] the target population was mainly constituted by patients with hypoproteinema, and diuretics were used, possibly interfering with the results. However, the trial from Martin and colleagues in 2005 [28] shows superiority of combined albumin and furosemide versus furosemide alone, and this mainly accounts for the positive results described earlier by this group [29]. In addition, all included studies used the ALI/ARDS criteria defined by the first consensus conference [15]. However, interpreting the results using the Berlin definition of ARDS [16] will result in a population of mild-to-moderate ARDS patients enrolled in the trials of Martin and colleagues [28,29], and moderate-to-severe ARDS in the SAFE trial [30]. Last but not least, the overall risk of bias was unclear, which may limit the interpretation of the results.

The strengths of this systematic review include the comprehensive search strategy using four databases, explicit inclusion and exclusion criteria, and the Cochrane risk of bias assessment [17] for each outcome and overall for each trial. Trial selection, data abstraction and risk of bias assessment were performed in duplicate. This systematic review is reported according to PRISMA guidelines [13].

This systematic review and meta-analysis may have implications for future RCTs. The meta-analyzed data suggest that patients with ARDS may be favored by albumin as compared to crystalloid solutions. Thus, in our opinion, a large multicenter trial investigating the effects of albumin solutions, or even a synthetic colloid, on lung function and damage in patients suffering from ARDS seems justifiable. Such a trial could focus on patients with ARDS of septic and non-septic origins since its presence can affect the patient outcome [36]. Ideally, the trial should be double-blinded, using different types of colloids (albumin, hydroxyethylstarch gelatin) as one group compared to crystalloids. Furthermore, the beginning of the intervention should be in the early stage of the disease and account for potential initial fluid resuscitation. Also, the hemodynamics variables should as closely as possible reflect the needs of ARDS patients, bearing in mind that we should not induce over-infusion. Certainly, the primary endpoint and secondary outcome should be hospital mortality, and lung function, respectively.

## Conclusion

From the present systematic review and meta-analysis we conclude that in patients with ARDS, therapy with albumin solutions improved the early oxygenation without affecting mortality, as compared to crystalloid solutions. Clearly, there is a need for large RCTs addressing the potential benefits of albumin solutions, or even synthetic colloids, as volume expanders in ARDS patients.

## Key messages

• Only three trials have compared colloid therapy to crystalloid therapy in ARDS patients.

• All these trials compared albumin to saline.

• Combined results of these studies showed no effect of albumin therapy on mortality, but showed an improvement in oxygenation.

• Given the small sample size and limited data on outcome, potential benefits of albumin solutions in ARDS patients remain uncertain.

## Abbreviations

ALI: acute lung injury; ARDS: acute respiratory distress syndrome; IL-8: interleukin 8; NGAL: neutrophil gelatinase-associated lipocalin; PaCO2: arterial carbon dioxide partial pressure; PaO2/FiO2: ratio of arterial partial pressure of oxygen/fraction of inspired oxygen; PEEP: positive end-expiratory pressure; PICOS: patient, population or problem, intervention, comparison, outcomes and setting; PRISMA: preferred reporting items for systematic reviews and meta-analyses; RCT: randomized controlled trial; RR: relative risk; SAFE trial: the saline versus albumin fluid evaluation trial; VE: vascular endothelial; WMD: weighted mean difference.

## Competing interests

The authors declare that they have no competing interests.

## Authors’ contributions

All authors developed the systematic review protocol. CU, PLS selected the articles and abstracted the data. SD designed the search strings. JS performed the statistical analysis. CU, PLS, and JS drafted the manuscript. MGA conceived the study and drafted the manuscript. All authors read and approved the final manuscript.

## Supplementary Material

Additional file 1 **Search strings.** This file contains the search strings. Click here for file

Additional file 2 **Table of excluded articles.** This file contains a table with the full-text articles excluded and respective reasons. Click here for file

## References

[B1] Matthay MA Ware LB Zimmerman GA The acute respiratory distress syndrome J Clin Invest 2012 122 2731 2740 10.1172/JCI60331 22850883 PMC3408735

[B2] Briel M Meade M Mercat A Brower RG Talmor D Walter SD Slutsky AS Pullenayegum E Zhou Q Cook D Brochard L Richard JC Lamontagne F Bhatnagar N Stewart TE Guyatt G Higher vs lower positive end-expiratory pressure in patients with acute lung injury and acute respiratory distress syndrome: systematic review and meta-analysis JAMA 2010 303 865 873 10.1001/jama.2010.218 20197533

[B3] Verheij J van Lingen A Raijmakers PG Rijnsburger ER Veerman DP Wisselink W Girbes AR Groeneveld AB Effect of fluid loading with saline or colloids on pulmonary permeability, oedema and lung injury score after cardiac and major vascular surgery Br J Anaesth 2006 96 21 30 16311279 10.1093/bja/aei286

[B4] Margarido CB Margarido NF Otsuki DA Fantoni DT Marumo CK Kitahara FR Magalhaes AA Pasqualucci CA Auler JO Jr Pulmonary function is better preserved in pigs when acute normovolemic hemodilution is achieved with hydroxyethyl starch versus lactated Ringer's solution Shock 2007 27 390 396 10.1097/01.shk.0000245026.01365.55 17414421

[B5] Di Filippo A Ciapetti M Prencipe D Tini L Casucci A Ciuti R Messeri D Falchi S Dani C Experimentally-induced acute lung injury: the protective effect of hydroxyethyl starch Ann Clin Lab Sci 2006 36 345 352 16951278

[B6] Huang CC Kao KC Hsu KH Ko HW Li LF Hsieh MJ Tsai YH Effects of hydroxyethyl starch resuscitation on extravascular lung water and pulmonary permeability in sepsis-related acute respiratory distress syndrome Crit Care Med 2009 37 1948 1955 10.1097/CCM.0b013e3181a00268 19384203

[B7] Camacho MT Totapally BR Torbati D Wolfsdorf J Pulmonary and extrapulmonary effects of increased colloid osmotic pressure during endotoxemia in rats Chest 2001 120 1655 1662 10.1378/chest.120.5.1655 11713150

[B8] Brunkhorst FM Engel C Bloos F Meier-Hellmann A Ragaller M Weiler N Moerer O Gruendling M Oppert M Grond S Olthoff D Jaschinski U John S Rossaint R Welte T Schaefer M Kern P Kuhnt E Kiehntopf M Hartog C Natanson C Loeffler M Reinhart K German Competence Network Sepsis (SepNet) Intensive insulin therapy and pentastarch resuscitation in severe sepsis N Engl J Med 2008 358 125 139 10.1056/NEJMoa070716 18184958

[B9] Perner A Haase N Wetterslev J Aneman A Tenhunen J Guttormsen AB Klemenzson G Pott F Bodker KD Badstolokken PM Bendtsen A Soe-Jensen P Tousi H Bestle M Pawlowicz M Winding R Bulow HH Kancir C Steensen M Nielsen J Fogh B Madsen KR Larsen NH Carlsson M Wiis J Petersen JA Iversen S Schoidt O Leivdal S Scandinavian Critical Care Trials Group Comparing the effect of hydroxyethyl starch 130/0.4 with balanced crystalloid solution on mortality and kidney failure in patients with severe sepsis (6S--Scandinavian Starch for Severe Sepsis/Septic Shock trial): study protocol, design and rationale for a double-blinded, randomised clinical trial Trials 2011 12 24-6215-12-24 21269526 10.1186/1745-6215-12-24 PMC3040153

[B10] Thomas-Rueddel DO Vlasakov V Reinhart K Jaeschke R Rueddel H Hutagalung R Stacke A Hartog CS Safety of gelatin for volume resuscitation–a systematic review and meta-analysis Intensive Care Med 2012 38 1134 1142 10.1007/s00134-012-2560-x 22527076

[B11] Perel P Roberts I Ker K Colloids versus crystalloids for fluid resuscitation in critically ill patients Cochrane Database Syst Rev 2013 2 CD000567 23450531 10.1002/14651858.CD000567.pub6

[B12] Reinhart K Perner A Sprung CL Jaeschke R Schortgen F Johan Groeneveld AB Beale R Hartog CS European Society of Intensive Care Medicine Consensus statement of the ESICM task force on colloid volume therapy in critically ill patients Intensive Care Med 2012 38 368 383 10.1007/s00134-012-2472-9 22323076

[B13] Moher D Liberati A Tetzlaff J Altman DG PRISMA Group Preferred reporting items for systematic reviews and meta-analyses: the PRISMA statement BMJ 2009 339 b2535 10.1136/bmj.b2535 19622551 PMC2714657

[B14] Riva JJ Malik KM Burnie SJ Endicott AR Busse JW What is your research question? An introduction to the PICOT format for clinicians J Can Chiropr Assoc 2012 56 167 171 22997465 PMC3430448

[B15] Bernard GR Artigas A Brigham KL Carlet J Falke K Hudson L Lamy M Legall JR Morris A Spragg R The American-European Consensus Conference on ARDS Definitions, mechanisms, relevant outcomes, and clinical trial coordination Am J Respir Crit Care Med 1994 149 818 824 10.1164/ajrccm.149.3.7509706 7509706

[B16] Ranieri VM Rubenfeld GD Thompson BT Ferguson ND Caldwell E Fan E Camporota L Slutsky AS ARDS Definition Task Force Acute respiratory distress syndrome: the Berlin Definition JAMA 2012 307 2526 2533 22797452 10.1001/jama.2012.5669

[B17] Higgins JP Altman DG Gotzsche PC Juni P Moher D Oxman AD Savovic J Schulz KF Weeks L Sterne JA Cochrane Bias Methods Group. Cochrane Statistical Methods Group The Cochrane Collaboration's tool for assessing risk of bias in randomised trials BMJ 2011 343 d5928 10.1136/bmj.d5928 22008217 PMC3196245

[B18] Guyatt GH Oxman AD Vist GE Kunz R Falck-Ytter Y Alonso-Coello P Schunemann HJ GRADE Working Group GRADE: an emerging consensus on rating quality of evidence and strength of recommendations BMJ 2008 336 924 926 10.1136/bmj.39489.470347.AD 18436948 PMC2335261

[B19] DerSimonian R Laird N Meta-analysis in clinical trials Control Clin Trials 1986 7 177 188 10.1016/0197-2456(86)90046-2 3802833

[B20] Zhang JC Ren HS Jiang JJ Ding M Meng M Zeng J Chu YF Zhu WY Qi GQ Wang P Wang CT The effects of joint administration of 6% hydroxyethyl starch 130/0.4 and high-volume hemofiltration on patients with acute lung injury and acute kidney injury Zhongguo Wei Zhong Bing Ji Jiu Yi Xue 2011 23 755 758 22153015

[B21] Khosropour R Lackner F Steinbereithner K Watzek C Piza F Wagner O Amesberger C Comparison of effects of hydroxyethylstarch (HES 200/0.5) administered pre- and postoperatively in vascular surgery with dextran 40 (60) (author's transl) Anaesthesist 1980 29 616 622 6161557

[B22] Coimbra VR Lara Rde A Flores EG Nozawa E Auler JO Jr Feltrim MI Application of noninvasive ventilation in acute respiratory failure after cardiovascular surgery Arq Bras Cardiol 2007 89 270 276 298–305 18066449 10.1590/s0066-782x2007001700004

[B23] Molnar Z Mikor A Leiner T Szakmany T Fluid resuscitation with colloids of different molecular weight in septic shock Intensive Care Med 2004 30 1356 1360 15127186 10.1007/s00134-004-2278-5

[B24] Xie J Yang J Effect of continuous high-volume hemofiltration on patients with acute respiratory distress syndrome and multiple organ dysfunction syndrome Zhongguo Wei Zhong Bing Ji Jiu Yi Xue 2009 21 402 404 19615130

[B25] Hankeln K Radel C Beez M Laniewski P Bohmert F Comparison of hydroxyethyl starch and lactated Ringer's solution on hemodynamics and oxygen transport of critically ill patients in prospective crossover studies Crit Care Med 1989 17 133 135 10.1097/00003246-198902000-00005 2464457

[B26] Hauser CJ Shoemaker WC Turpin I Goldberg SJ Oxygen transport responses to colloids and crystalloids in critically ill surgical patients Surg Gynecol Obstet 1980 150 811 816 7376041

[B27] Quinlan GJ Mumby S Martin GS Bernard GR Gutteridge JM Evans TW Albumin influences total plasma antioxidant capacity favorably in patients with acute lung injury Crit Care Med 2004 32 755 759 10.1097/01.CCM.0000114574.18641.5D 15090958

[B28] Martin GS Moss M Wheeler AP Mealer M Morris JA Bernard GR A randomized, controlled trial of furosemide with or without albumin in hypoproteinemic patients with acute lung injury Crit Care Med 2005 33 1681 1687 10.1097/01.CCM.0000171539.47006.02 16096441

[B29] Martin GS Mangialardi RJ Wheeler AP Dupont WD Morris JA Bernard GR Albumin and furosemide therapy in hypoproteinemic patients with acute lung injury Crit Care Med 2002 30 2175 2182 10.1097/00003246-200210000-00001 12394941

[B30] Finfer S Bellomo R Boyce N French J Myburgh J Norton R SAFE Study Investigators A comparison of albumin and saline for fluid resuscitation in the intensive care unit N Engl J Med 2004 350 2247 2256 15163774 10.1056/NEJMoa040232

[B31] Bhattacharya J Matthay MA Regulation and repair of the alveolar-capillary barrier in acute lung injury Annu Rev Physiol 2013 75 593 615 10.1146/annurev-physiol-030212-183756 23398155

[B32] Herwig MC Tsokos M Hermanns MI Kirkpatrick CJ Muller AM Vascular endothelial cadherin expression in lung specimens of patients with sepsis-induced acute respiratory distress syndrome and endothelial cell cultures Pathobiology 2013 80 245 251 10.1159/000347062 23635392

[B33] Leung AD Wong KH Tien J Plasma expanders stabilize human microvessels in microfluidic scaffolds J Biomed Mater Res A 2012 100 1815 1822 22489049 10.1002/jbm.a.34137 PMC3360840

[B34] Moriondo A Pelosi P Passi A Viola M Marcozzi C Severgnini P Ottani V Quaranta M Negrini D Proteoglycan fragmentation and respiratory mechanics in mechanically ventilated healthy rats J Appl Physiol 2007 103 747 756 10.1152/japplphysiol.00056.2007 17569774

[B35] Myburgh JA Finfer S Bellomo R Billot L Cass A Gattas D Glass P Lipman J Liu B McArthur C McGuinness S Rajbhandari D Taylor CB Webb SA CHEST Investigators, Australian and New Zealand Intensive Care Society Clinical Trials Group Hydroxyethyl starch or saline for fluid resuscitation in intensive care N Engl J Med 2012 367 1901 1911 10.1056/NEJMoa1209759 23075127

[B36] Sheu CC Gong MN Zhai R Chen F Bajwa EK Clardy PF Gallagher DC Thompson BT Christiani DC Clinical characteristics and outcomes of sepsis-related vs non-sepsis-related ARDS Chest 2010 138 559 567 10.1378/chest.09-2933 20507948 PMC2940067

