# Supplementary material for: Albumin versus crystalloid solutions in patients with the acute respiratory distress syndrome: a systematic review and meta-analysis
Source: Crit Care. 2014 Jan 9;18(1):R10. doi: 10.1186/cc13187 (PMC4056106; doi:10.1186/cc13187)
Supplement: Additional file 1 — Search strings. This file contains the search strings. [file cc13187-S1.docx]

**Additional File 1: Search Strings**

**MEDLINE via Pubmed**

#1

((randomized controlled trial[pt]) OR (controlled clinical trial[pt]) OR (randomized[tiab]) OR (placebo[tiab]) OR (clinical trials as topic[mesh:noexp]) OR (randomly[tiab]) OR (trial[ti])) NOT (animals[mh] NOT (humans[mh] AND animals[mh]))

#2

((critically ill) OR (respiratory drive[tw]) OR (respiratory depression) OR (respiratory failure) OR (Lung Injury[MeSH Terms]) OR (Lung Diseases[MeSH Terms]) OR (respiratory distress[tw]) OR (ARDS[tw]) OR (Respiratory Distress Syndrome,Adult[MH]))

#3

((Plasma Volume[MeSH Terms]) OR (fluid therapy[MeSH Terms]) OR (colloids[MeSH Terms]) OR (resuscitation[MeSH Terms]) OR (hetastarch[MeSH Terms]) OR (albumins[MeSH Terms]) OR (gelatin[MeSH Terms]) OR (dextrans[MeSH Terms]) OR (Rehydration Solutions[MeSH Terms]) OR (Isotonic Solutions[MeSH Terms]) OR (Plasma[MeSH Terms]) OR (Plasma Substitutes[MeSH Terms]) NOT (infants[MeSH Terms])))

#1 AND #2 AND #3

**CENTRAL**

#1

((randomized controlled trial):pt or (controlled clinical trial):pt or (randomized):ti,ab or (placebo):ti,ab or (clinical trials as topic):kw or (randomly):ti,ab or (trial):ti) not (animals:kw not (humans and animals):kw)

#2

((critically ill) or (respiratory drive) or (respiratory depression) or (respiratory failure) or (Lung Injury):kw or (Lung Diseases):kw or (respiratory distress) or (ARDS) or (Respiratory Distress Syndrome,Adult))

#3

((Plasma Volume):kw or (fluid therapy):kw or (colloids):kw or (hydrocolloid):kw or (resuscitation):kw or (hetastarch):kw or (pentastarch):kw or (albumin):kw or (gelatin):kw or (dextran):kw or (Rehydration Solutions):kw or (Isotonic Solutions):kw or (Plasma):kw or (Plasma Substitutes):kw or (hydrocolloid):kw not (infants):kw)

(#1) and (#2) and (#3)

**EMBASE**

1 crossover-procedure/
2 double-blind procedure/
3 randomised controlled trial/
4 single-blind procedure/
5 (random$ or factorial$ or crossover$ or cross over$ or cross-over$ or placebo$ or (doubl$ adj blind$) or (singl$ adj blind$) or assign$ or allocat$ or volunteer$).tw.
6 or/1-5
7 exp animals/
8 exp humans/
9 7 not (7 and 8)
10 6 not 9
11 (critically ill or respiratory drive or respiratory depression or respiratory failure or ARDS).mp. or (Lung Injury or Lung Disease or respiratory distress or adult respiratory distress syndrome).sh.
12 and/10-11
13 ((Plasma Volume or fluid therapy or colloid or hydrocolloid or resuscitation or hetastarch or albumin or gelatin or dextran or oral Rehydration Solution or Isotonic Solution or Plasma or Plasma Substitute).sh. or pentastarch.mp.) not infant.sh.
13 and/12-13

**LILACS**

#1
((PT:"randomized controlled trial" OR PT:"controlled clinical trial" OR PT:"multicenter study" OR MH:"randomized controlled trials as topic" OR MH:"controlled clinical trials as topic" OR MH:"multicenter studies as topic" OR MH:"random allocation" OR MH:"double-blind method" OR MH:"single-blind method") OR ((ensaio$ OR ensayo$ OR trial$) AND (azar OR acaso OR placebo OR control$ OR aleat$ OR random$ OR enmascarado$ OR simpleciego OR ((simple$ OR single OR duplo$ OR doble$ OR double$) AND (cego OR ciego OR blind OR mask))) AND clinic$)) AND NOT (MH:animals OR MH:rabbits OR MH:rats OR MH:primates OR MH:dogs OR MH:cats OR MH:swine OR PT:"in vitro")

#2

(critically ill OR respiratory drive OR TW:respiratory depression OR respiratory failure OR Lung Injury OR MH:Lung Diseases OR ARDS OR MH:Adult Respiratory Distress Syndrome)

#3)
(MH:Plasma Volume OR MH:fluid therapy OR MH:colloids OR hydrocolloid OR MH:resuscitation OR MH:hetastarch OR TW:pentastarch OR MH:albumin OR MH:gelatin OR MH:dextran OR MH:Rehydration Solutions OR MH:Isotonic Solutions OR Plasma OR MH:Plasma Substitutes)

#4 NOT infants

#1 AND #2 AND #3 AND #4
