# Supplementary material for: Albumin versus crystalloid solutions in patients with the acute respiratory distress syndrome: a systematic review and meta-analysis
Source: Crit Care. 2014 Jan 9;18(1):R10. doi: 10.1186/cc13187 (PMC4056106; doi:10.1186/cc13187)
Supplement: Additional file 2 — Table of excluded articles. This file contains a table with the full-text articles excluded and respective reasons. [file cc13187-S2.docx]

**Additional File 2: Table of Full-text Articles Excluded, with Reasons**

| **First Author** | **Title** | **Reason for Exclusion** |
| --- | --- | --- |
| Barquist 1999 [1] | A randomized prospective trial of amphotericin B lipid emulsion versus dextrose colloidal solution in critically ill patients | Population: no ARDS patients |
| Beards 1994 [2] | Comparison of the hemodynamic and oxygen transport responses to modified fluid gelatin and hetastarch in critically ill patients: a prospective, randomized trial | Population: no ARDS patients |
| Bellomo 2006 [3] | The effects of saline or albumin resuscitation on acid-base status and serum electrolytes | Population: no ARDS patients |
| Bellomo 2009 [4] | Effects of saline or albumin resuscitation on standard coagulation tests | Population: no ARDS patients |
| Bentsen 2006 [5] | Hypertonic saline (7.2%) in 6% hydroxyethyl starch reduces intracranial pressure and improves hemodynamics in a placebo-controlled study involving stable patients with subarachnoid hemorrhage. | Population: no ARDS patients |
| Bickell 1994 [6] | Immediate versus delayed fluid resuscitation for hypotensive patients with penetrating torso injuries | Population: no ARDS patients |
| Bland 1976 [7] | Rapid infusion of sodium bicarbonate and albumin into high-risk premature infants soon after birth: a controlled, prospective trial. | Population: no ARDS patients |
| Boldt 1995 [8] | Does the type of volume therapy influence endothelial-related coagulation in the critically ill? | Population: no ARDS patients |
| Boldt 1996 [9] | Influence of different volume therapy regimens on regulators of the circulation in the critically ill | Population: no ARDS patients |
| Boldt 1996 [10] | Influence of different volume therapies on platelet function in the critically ill | Population: no ARDS patients |
| Boldt 1998 [11] | Volume therapy in the critically ill: is there a difference? | Population: no ARDS patients |
| Boldt 1996 [12] | The effects of albumin versus hydroxyethyl starch solution on cardiorespiratory and circulatory variables in critically ill patients | Population: no ARDS patients |
| Brunkhorst 2008 [13] | Intensive insulin therapy and pentastarch resuscitation in severe sepsis. | Population: no ARDS patients |
| Bueno 2004 [14] | Effects of hypertonic saline-dextran solution in cardiac valve surgery with cardiopulmonary bypass | Population: no ARDS patients |
| Buhre 1997 [15] | Acute effect of mitral calve replacement on extravascular lung water in patients receiving colloid or crystalloid priming of cardiopulmonary bypass | Population: no ARDS patients |
| Bulger 2008 [16] | Hypertonic resuscitation of hypovolemic shock after blunt trauma: a randomized controlled trial | Population: no ARDS patients |
| Chodri 2003 [17] | Albumin infusion with diuretics, a controversy revisited. | Study: no RCT |
| Coimbra 2007 [18] | Application of noninvasive ventilation in acute respiratory failure after cardiovascular surgery | Comparison: no colloids used |
| Davies 1979 [18] | South Wales multicenter trial of prophylactic Dextran 70 after surgery: a clinically oriented randomized double-blind trial. Further observations | Population: no ARDS patients |
| Dolecek 2009 [19] | Therapeutic influence of 20 % albumin versus 6% hydroxyethylstarch on extravascular lung water in septic patients: a randomized controlled trial | Population: no ARDS patients |
| Du, X 2011 [20] | Hydroxyethyl starch resuscitation reduces the risk of intra-abdominal hypertension in severe acute pancreatitis. | Population: no ARDS patients |
| Dubois 2006 [21] | Albumin administration improves organ function in critically ill hypoalbuminemic patients: A prospective, randomized, controlled, pilot study | Population: no ARDS patients |
| Ernest 1999 [22] | Distribution of normal saline and 5% albumin infusions in septic patients. | Population: no ARDS patients |
| Foley 1990 [23] | Albumin supplementation in the critically ill. A prospective, randomized trial. | Population: no ARDS patients |
| Forget 2010 [24] | Goal-directed fluid management based on the pulse oximeter-derived pleth variability index reduces lactate levels and improves fluid management. | Population: no ARDS patients |
| Golub 1994 [25] | Efficacy of albumin supplementation in the surgical intensive care unit: a prospective, randomized study | Population: no ARDS patients |
| Gondos T 2010 [26] | Short-term effectiveness of different volume replacement therapies in postoperative hypovolaemic patients | Population: no ARDS patients |
| Hankeln 1989 [27] | Comparison of hydroxyethyl starch and lactacted Ringer's solution on hemodynamics and oxygen transport of critically ill patients in prospective crossover studies. | Outcome: only two ARDS patients, only functional data reported |
| Hauser 1980 [28] | Oxygen transport responses to colloids and crystalloids in critically ill surgical patients. | Outcome: only one secondary outcome |
| Heijden 2009 [29] | Crystalloid or colloid fluid loading and pulmonary permeability, edema, and injury in septic and nonseptic critically ill patients with hypovolemia. | Population: no ARDS patients |
| Khosropour 1980 [30] | Comparison of effects of hydroxyethylstarch (HES 200/0.5) administered pre- and postoperatively in vascular surgery with dextrane 40 (60) | Population: no ARDS patients |
| Krasheninnikov 2007 [31] | Effect of various colloidal solutions on pulmonary oxygenizing function in patients with acute lung lesion | Population: no ARDS patients |
| Kuper 2007 [32] | The short-term effect of hyperoncotic albumin, given alone or with furosemide, on oxygenation in sepsis-induced acute respiratory distress syndrome. | Study: no RCT |
| Lazrove 1980[33] | Hemodynamic, blood volume, and oxygen transport responses to albumin and hydroxyethyl starch infusions in critically ill postoperative patients | Population: no ARDS patients |
| Lowe 1977 [34] | Crystalloid vs colloid in the etiology of pulmonary failure after trauma: a randomized trial in man. | Population: no ARDS patients |
| Mahmood 2009 [35] | Splanchnic Microcirculation Protection by Hydroxyethyl Starches During Abdominal Aortic Aneurysm Surgery. | Population: no ARDS patients |
| Maitland 2011 [36] | Mortality after fluid bolus in African children with severe infection. | Population: no ARDS patients |
| Martin 2002 [37] | Findings on the portable chest radiograph correlate with fluid balance in critically ill patients | Study: ancillary study of a RCT |
| Mbaba Mena 2000 [38] | Effects of a hydroxyethylstarch solution on plasma colloid osmotic pressure in acutely ill patients. | Population: no ARDS patients |
| Mellbring 1986 [39] | Venous thromboembolism after cerebral infarction and the prophylactic effect of dextran 40 | Population: no ARDS patients |
| Metildi 1984 [40] | Crystalloid versus colloid in fluid resuscitation of patients with severe pulmonary insufficiency | Population: no ARDS patients |
| Modig 1983 [41] | Advantages of dextran 70 over Ringer acetate solution in shock treatment and in prevention of adult respiratory distress syndrome. A randomized study in man after traumatic-haemorrhagic shock | Population: no ARDS patients |
| Modig 1986 [42] | Effectiveness of dextran 70 versus Ringer's acetate in traumatic shock and adult respiratory distress syndrome | Population: no ARDS patients |
| Molnar 2004 [43] | Fluid resuscitation with colloids of different molecular weight in septic shock | Comparison: both groups received colloids |
| Moreau 2006 [44] | Comparison of outcome in patients with cirrhosis and ascites following treatment with albumin or a synthetic colloid: A randomised controlled pilot trial. | Population: no ARDS patients |
| Myburgh 2007 [45] | Saline or albumin for fluid resuscitation in patients with traumatic brain injury. | Study: no RCT, subgroup analysis SAFE Study |
| Myburgh 2012 [46] | Hydroxyethyl starch or saline for fluid resuscitation in intensive care | Population: no ARDS patients |
| Neff 2003 [47] | Repetitive large-dose infusion of the novel hydroxyethyl starch 130/0.4 in patients with severe head injury. | Population: no ARDS patients |
| Nillius 1979 [48] | Preoperative normovolaemic haemodilution with dextran 70 as a thromboembolic prophylaxis in total in hip replacement | Population: no ARDS patients |
| Oertli 1992 [49] | Prevention of deep vein thrombosis in patients with hip fractures: low molecular weight heparin versus dextran | Population: no ARDS patients |
| Palumbo 2006 [50] | The effects of hydroxyethyl starch solution in critically ill patients. | Population: no ARDS patients |
| Quinlan 2004 [51] | Albumin influences total plasma antioxidant capacity favorably in patients with acute lung injury | Outcome: none of the outcomes measured |
| Reed 1985 [52] | Dextran 70 versus donor plasma as colloid in open-heart surgery under extreme haemodilution. | Population: no ARDS patients |
| Rittoo 2004 [53] | Randomized study comparing the effects of hydroxyethyl starch solution with Gelofusine on pulmonary function in patients undergoing abdominal aortic aneurysm surgery. | Population: no ARDS patients |
| Senagore 2009 [54] | Fluid management for laparoscopic colectomy: A prospective, randomized assessment of goal-directed administration of balanced salt solution or hetastarch coupled with an enhanced recovery program. | Population: no ARDS patients |
| Smith 1978 [55] | Dextran and intermittent pneumatic compression in prevention of postoperative deep vein thrombosis: multiunit trial | Population: no ARDS patients |
| Stockwell 1992 [56] | Colloid solutions in the critically ill. A randomised comparison of albumin and polygeline. 1. Outcome and duration of stay in the intensive care unit. | Population: no ARDS patients |
| Trof 2010 [57] | Greater cardiac response of colloid than saline fluid loading in septic and non-septic critically ill patients with clinical hypovolaemia. | Population: no ARDS patients |
| Turner 2000 [58] | A randomised controlled trial of prehospital intravenous fluid replacement therapy in serious trauma. | Population: no ARDS patients |
| Veneman 2004 [59] | Human albumin and starch administration in critically ill patients: a prospective randomized clinical trial. | Population: no ARDS patients |
| Verheij 2006 [60] | Effect of fluid loading with saline or colloids on pulmonary permeability, oedema and lung injury score after cardiac and major vascular surgery. | Population: no ARDS patients |
| Wahba 1996 [61] | Fluid resuscitation with Haemaccel (R) vs. human albumin following coronary artery bypass grafting | Population: no ARDS patients |
| Xie 2009 [62] | Effect of continuous high-volume hemofiltration on patients with acute respiratory distress syndrome and multiple organ dysfunction syndrome | Comparison: no colloids used |
| Yu 1995 [63] | Frequency of mortality and myocardial infarction during maximizing oxygen delivery: a prospective, randomized trial. | Population: no ARDS patients |
| Yu 2011 [64] | A prospective randomized trial using blood volume analysis in addition to pulmonary artery catheter, compared with pulmonary artery catheter alone, to guide shock resuscitation in critically ill surgical patients. | Population: no ARDS patients |
| Zhang 2011 [65] | The effects of joint administration of 6% hydroxyethyl starch 130/0.4 and high-volume hemofiltration on patients with acute lung injury and acute kidney injury | Comparison: both groups received colloids |

ARDS = acute respiratory distress syndrome, RCT = randomized controlled trial

**References**

1. Barquist E, Fein E, Shadick D, Johnson J, Clark J, Shatz D: **A randomized prospective trial of amphotericin B lipid emulsion versus dextrose colloidal solution in critically ill patients.** *J Trauma* 1999, **47**(2):336-340.

2. Beards SC, Watt T, Edwards JD, Nightingale P, Farragher EB: **Comparison of the hemodynamic and oxygen transport responses to modified fluid gelatin and hetastarch in critically ill patients: a prospective, randomized trial.** *Crit Care Med* 1994, **22**(4):600-605.

3. Bellomo R, Morimatsu H, French C, Cole L, Story D, Uchino S, Naka T, SAFE Study Investigators: **The effects of saline or albumin resuscitation on acid-base status and serum electrolytes.** *Crit Care Med* 2006, **34**(12):2891-2897.

4. Bellomo R, Morimatsu H, Presneill J, French C, Cole L, Story D, Uchino S, Naka T, Finfer S, Cooper DJ, Myburgh J, SAFE Study Investigators and the Australian and New Zealand Intensive Care Society Clinical Trials Group: **Effects of saline or albumin resuscitation on standard coagulation tests.** *Crit Care Resusc* 2009, **11**(4):250-256.

5. Bentsen G, Breivik H, Lundar T, Stubhaug A: **Hypertonic saline (7.2%) in 6% hydroxyethyl starch reduces intracranial pressure and improves hemodynamics in a placebo-controlled study involving stable patients with subarachnoid hemorrhage.** *Crit Care Med* 2006, **34**(12):2912-2917.

6. Bickell WH, Wall MJ,Jr, Pepe PE, Martin RR, Ginger VF, Allen MK, Mattox KL: **Immediate versus delayed fluid resuscitation for hypotensive patients with penetrating torso injuries.** *N Engl J Med* 1994, **331**(17):1105-1109.

7. Bland RD, Clarke TL, Harden LB: **Rapid infusion of sodium bicarbonate and albumin into high-risk premature infants soon after birth: a controlled, prospective trial.** *Am J Obstet Gynecol* 1976, **124**(3):263-267.

8. Boldt J, Heesen M, Welters I, Padberg W, Martin K, Hempelmann G: **Does the type of volume therapy influence endothelial-related coagulation in the critically ill?** *Br J Anaesth* 1995, **75**(6):740-746.

9. Boldt J, Mueller M, Menges T, Papsdorf M, Hempelmann G: **Influence of different volume therapy regimens on regulators of the circulation in the critically ill.** *Br J Anaesth* 1996, **77**(4):480-487.

10. Boldt J, Muller M, Heesen M, Heyn O, Hempelmann G: **Influence of different volume therapies on platelet function in the critically ill.** *Intensive Care Med* 1996, **22**(10):1075-1081.

11. Boldt J, Muller M, Mentges D, Papsdorf M, Hempelmann G: **Volume therapy in the critically ill: is there a difference?** *Intensive Care Med* 1998, **24**(1):28-36.

12. Boldt J, Heesen M, Muller M, Pabsdorf M, Hempelmann G: **The effects of albumin versus hydroxyethyl starch solution on cardiorespiratory and circulatory variables in critically ill patients.** *Anesth Analg* 1996, **83**(2):254-261.

13. Brunkhorst FM, Engel C, Bloos F, Meier-Hellmann A, Ragaller M, Weiler N, Moerer O, Gruendling M, Oppert M, Grond S, Olthoff D, Jaschinski U, John S, Rossaint R, Welte T, Schaefer M, Kern P, Kuhnt E, Kiehntopf M, Hartog C, Natanson C, Loeffler M, Reinhart K, German Competence Network Sepsis (SepNet): **Intensive insulin therapy and pentastarch resuscitation in severe sepsis.** *N Engl J Med* 2008, **358**(2):125-139.

14. Bueno R, Resende AC, Melo R, Neto VA, Stolf NA: **Effects of hypertonic saline-dextran solution in cardiac valve surgery with cardiopulmonary bypass.** *Ann Thorac Surg* 2004, **77**(2):604-11; discussion 611.

15. Buhre W, Hoeft A, Schorn B, Weyland A, Scholz M, Sonntag H: **Acute affect of mitral calve replacement on extravascular lung water in patients receiving colloid or crystalloid priming of cardiopulmonary bypass.** *Br J Anaesth* 1997, **79**(3):311-316.

16. Bulger EM, Jurkovich GJ, Nathens AB, Copass MK, Hanson S, Cooper C, Liu PY, Neff M, Awan AB, Warner K, Maier RV: **Hypertonic resuscitation of hypovolemic shock after blunt trauma: a randomized controlled trial.** *Arch Surg* 2008, **143**(2):139-48; discussion 149.

17. Chodri TA, Groth ML: **Albumin infusion with diuretics, a controversy revisited.** *Clinical Pulmonary Medicine* 2003, **10**(2):121-123.

18. Coimbra VR, Lara Rde A, Flores EG, Nozawa E, Auler Jr JO, Feltrim MI: **Application of noninvasive ventilation in acute respiratory failure after cardiovascular surgery.** *Arq Bras Cardiol* 2007, **89**(5):270-6, 298-305.

19. Dolecek M, Svoboda P, Kantorova I, Scheer P, Sas I, Bibrova J, Radvanova J, Radvan M: **Therapeutic influence of 20 % albumin versus 6% hydroxyethylstarch on extravascular lung water in septic patients: a randomized controlled trial.** *Hepatogastroenterology* 2009, **56**(96):1622-1628.

20. Du X-, Hu W-, Xia Q, Huang Z-, Chen G-, Jin X-, Xue P, Lu H-, Ke N-, Zhang Z-, Li Q-: **Hydroxyethyl starch resuscitation reduces the risk of intra-abdominal hypertension in severe acute pancreatitis.** *Pancreas* 2011, **40**(8):1220-1225.

21. Dubois MJ, Orellana-Jimenez C, Melot C, De Backer D, Berre J, Leeman M, Brimioulle S, Appoloni O, Creteur J, Vincent JL: **Albumin administration improves organ function in critically ill hypoalbuminemic patients: A prospective, randomized, controlled, pilot study.** *Crit Care Med* 2006, **34**(10):2536-2540.

22. Ernest D, Belzberg AS, Dodek PM: **Distribution of normal saline and 5% albumin infusions in septic patients.** *Crit Care Med* 1999, **27**(1):46-50.

23. Foley EF, Borlase BC, Dzik WH, Bistrian BR, Benotti PN: **Albumin supplementation in the critically ill. A prospective, randomized trial.** *Arch Surg* 1990, **125**(6):739-742.

24. Forget P, Lois F, De Kock M: **Goal-directed fluid management based on the pulse oximeter-derived pleth variability index reduces lactate levels and improves fluid management.** *Anesth Analg* 2010, **111**(4):910-914.

25. Golub R, Sorrento JJ,Jr, Cantu R,Jr, Nierman DM, Moideen A, Stein HD: **Efficacy of albumin supplementation in the surgical intensive care unit: a prospective, randomized study.** *Crit Care Med* 1994, **22**(4):613-619.

26. Gondos T, Marjanek Z, Ulakcsai Z, Szabo Z, Bogar L, Karolyi M, Gartner B, Kiss K, Havas A, Futo J: **Short-term effectiveness of different volume replacement therapies in postoperative hypovolaemic patients.** *Eur J Anaesthesiol* 2010, **27**(9):794-800.

27. Hankeln K, Radel C, Beez M, Laniewski P, Bohmert F: **Comparison of hydroxyethyl starch and lactacted Ringer's solution on hemodynamics and oxygen transport of critically ill patients in prospective crossover studies.** *Crit Care Med* 1989, **17**(2):133-135.

28. Hauser CJ, Shoemaker WC, Turpin I, Goldberg SJ: **Oxygen transport responses to colloids and crystalloids in critically ill surgical patients.** *Surg Gynecol Obstet* 1980, **150**(6):811-816.

29. van der Heijden M, Verheij J, van Nieuw Amerongen GP, Groeneveld AB: **Crystalloid or colloid fluid loading and pulmonary permeability, edema, and injury in septic and nonseptic critically ill patients with hypovolemia.** *Crit Care Med* 2009, **37**(4):1275-1281.

30. Khosropour R, Lackner F, Steinbereithner K, Watzek C, Piza F, Wagner O, Amesberger C: **Comparison of effects of hydroxyethylstarch (HES 200/0.5) administered pre- and postoperatively in vascular surgery with dextran 40 (60) (author's transl).** *Anaesthesist* 1980, **29**(11):616-622.

31. Krasheninnikov SV, Levit AL, Leiderman IN, Malkova OG: **Effect of various colloidal solutions on pulmonary oxygenizing function in patients with acute lung lesion.** *Anesteziol Reanimatol* 2007, **(3)**(3):20-22.

32. Kuper M, Gunning MP, Halder S, Soni N: **The short-term effect of hyperoncotic albumin, given alone or with furosemide, on oxygenation in sepsis-induced acute respiratory distress syndrome.** *Anaesthesia* 2007, **62**(3):259-263.

33. Lazrove S, Waxman K, Shippy C, Shoemaker WC: **Hemodynamic, blood volume, and oxygen transport responses to albumin and hydroxyethyl starch infusions in critically ill postoperative patients.** *Crit Care Med* 1980, **8**(5):302-306.

34. Lowe RJ, Moss GS, Jilek J, Levine HD: **Crystalloid vs colloid in the etiology of pulmonary failure after trauma: a randomized trial in man.** *Surgery* 1977, **81**(6):676-683.

35. Mahmood A, Gosling P, Barclay R, Kilvington F, Vohra R: **Splanchnic Microcirculation Protection by Hydroxyethyl Starches During Abdominal Aortic Aneurysm Surgery.** *European Journal of Vascular and Endovascular Surgery* 2009, **37**(3):319-325.

36. Maitland K, Kiguli S, Opoka RO, Engoru C, Olupot-Olupot P, Akech SO, Nyeko R, Mtove G, Reyburn H, Lang T, Brent B, Evans JA, Tibenderana JK, Crawley J, Russell EC, Levin M, Babiker AG, Gibb DM: **Mortality after fluid bolus in African children with severe infection.** *N Engl J Med* 2011, **364**(26):2483-2495.

37. Martin GS, Ely EW, Carroll FE, Bernard GR: **Findings on the portable chest radiograph correlate with fluid balance in critically ill patients.** *Chest* 2002, **122**(6):2087-2095.

38. Mbaba Mena J, De Backer D, Vincent JL: **Effects of a hydroxyethylstarch solution on plasma colloid osmotic pressure in acutely ill patients.** *Acta Anaesthesiol Belg* 2000, **51**(1):39-42.

39. Mellbring G, Strand T, Eriksson S: **Venous thromboembolism after cerebral infarction and the prophylactic effect of dextran 40.** *Acta Med Scand* 1986, **220**(5):425-429.

40. Metildi LA, Shackford SR, Virgilio RW, Peters RM: **Crystalloid versus colloid in fluid resuscitation of patients with severe pulmonary insufficiency.** *Surg Gynecol Obstet* 1984, **158**(3):207-212.

41. Modig J: **Advantages of dextran 70 over Ringer acetate solution in shock treatment and in prevention of adult respiratory distress syndrome. A randomized study in man after traumatic-haemorrhagic shock.** *Resuscitation* 1983, **10**(4):219-226.

42. Modig J: **Effectiveness of dextran 70 versus Ringer's acetate in traumatic shock and adult respiratory distress syndrome.** *Crit Care Med* 1986, **14**(5):454-457.

43. Molnar Z, Mikor A, Leiner T, Szakmany T: **Fluid resuscitation with colloids of different molecular weight in septic shock.** *Intensive Care Med* 2004, **30**(7):1356-1360.

44. Moreau R, Valla DC, Durand-Zaleski I, Bronowicki JP, Durand F, Chaput JC, Dadamessi I, Silvain C, Bonny C, Oberti F, Gournay J, Lebrec D, Grouin JM, Guemas E, Golly D, Padrazzi B, Tellier Z: **Comparison of outcome in patients with cirrhosis and ascites following treatment with albumin or a synthetic colloid: a randomised controlled pilot trail.** *Liver Int* 2006, **26**(1):46-54.

45. Myburgh J, Cooper DJ, Finfer S, Bellomo R, Norton R, Bishop N, Kai Lo S, Vallance S: **Saline or albumin for fluid resuscitation in patients with traumatic brain injury.** *N Engl J Med* 2007, **357**(9):874-884.

46. Myburgh JA, Finfer S, Bellomo R, Billot L, Cass A, Gattas D, Glass P, Lipman J, Liu B, McArthur C, McGuinness S, Rajbhandari D, Taylor CB, Webb SA, CHEST Investigators, Australian and New Zealand Intensive Care Society Clinical Trials Group: **Hydroxyethyl starch or saline for fluid resuscitation in intensive care.** *N Engl J Med* 2012, **367**(20):1901-1911.

47. Neff TA, Doelberg M, Jungheinrich C, Sauerland A, Spahn DR, Stocker R: **Repetitive large-dose infusion of the novel hydroxyethyl starch 130/0.4 in patients with severe head injury.** *Anesth Analg* 2003, **96**(5):1453-9, table of contents.

48. Nillius SA, Ahlberg A, Arborelius M,Jr, Rosberg B: **Preoperative normovolaemic haemodilution with dextran 70 as a thromboembolic prophylaxis in total in hip replacement.** *Int Orthop* 1979, **3**(3):197-202.

49. Oertli D, Hess P, Durig M, Laffer U, Fridrich R, Jaeger K, Kaufmann R, Harder F: **Prevention of deep vein thrombosis in patients with hip fractures: low molecular weight heparin versus dextran.** *World J Surg* 1992, **16**(5):980-4; discussion 984-5.

50. Palumbo D, Servillo G, D'Amato L, Volpe ML, Capogrosso G, De Robertis E, Piazza O, Tufano R: **The effects of hydroxyethyl starch solution in critically ill patients.** *Minerva Anestesiol* 2006, **72**(7-8):655-664.

51. Quinlan GJ, Mumby S, Martin GS, Bernard GR, Gutteridge JM, Evans TW: **Albumin influences total plasma antioxidant capacity favorably in patients with acute lung injury.** *Crit Care Med* 2004, **32**(3):755-759.

52. Reed RK, Lilleaasen P, Lindberg H, Stokke O: **Dextran 70 versus donor plasma as colloid in open-heart surgery under extreme haemodilution.** *Scand J Clin Lab Invest* 1985, **45**(3):269-274.

53. Rittoo D, Gosling P, Burnley S, Bonnici C, Millns P, Simms MH, Smith SRG, Vohra RK: **Randomized study comparing the effects of hydroxyethyl starch solution with Gelofusine on pulmonary function in patients undergoing abdominal aortic aneurysm surgery.** *Br J Anaesth* 2004, **92**(1):61-66.

54. Senagore AJ, Emery T, Luchtefeld M, Kim D, Dujovny N, Hoedema R: **Fluid management for laparoscopic colectomy: A prospective, randomized assessment of goal-directed administration of balanced salt solution or hetastarch coupled with an enhanced recovery program.** *Dis Colon Rectum* 2009, **52**(12):1935-1940.

55. Smith RC, Elton RA, Orr JD, Hart AJ, Graham IF, Fuller GA, Rundle JS, Macpherson AI, Ruckley CV: **Dextran and intermittent pneumatic compression in prevention of postoperative deep vein thrombosis: multiunit trial.** *Br Med J* 1978, **1**(6118):952-954.

56. Stockwell MA, Scott A, Day A, Riley B, Soni N: **Colloid solutions in the critically ill. A randomised comparison of albumin and polygeline 2. Serum albumin concentration and incidences of pulmonary oedema and acute renal failure.** *Anaesthesia* 1992, **47**(1):7-9.

57. Trof RJ, Sukul SP, Twisk JW, Girbes AR, Groeneveld AB: **Greater cardiac response of colloid than saline fluid loading in septic and non-septic critically ill patients with clinical hypovolaemia.** *Intensive Care Med* 2010, **36**(4):697-701.

58. Turner J, Nicholl J, Webber L, Cox H, Dixon S, Yates D: **A randomised controlled trial of prehospital intravenous fluid replacement therapy in serious trauma.** *Health Technol Assess* 2000, **4**(31):1-57.

59. Veneman TF, Oude Nijhuis J, Woittiez AJ: **Human albumin and starch administration in critically ill patients: a prospective randomized clinical trial.** *Wien Klin Wochenschr* 2004, **116**(9-10):305-309.

60. Verheij J, van Lingen A, Raijmakers PG, Rijnsburger ER, Veerman DP, Wisselink W, Girbes AR, Groeneveld AB: **Effect of fluid loading with saline or colloids on pulmonary permeability, oedema and lung injury score after cardiac and major vascular surgery.** *Br J Anaesth* 2006, **96**(1):21-30.

61. Wahba A, Sendtner E, Birnbaum DE: **Fluid resuscitation with Haemaccel vs. human albumin following coronary artery bypass grafting.** *Thorac Cardiovasc Surg* 1996, **44**(4):178-182.

62. Xie J, Yang J: **Effect of continuous high-volume hemofiltration on patients with acute respiratory distress syndrome and multiple organ dysfunction syndrome.** *Zhongguo Wei Zhong Bing Ji Jiu Yi Xue* 2009, **21**(7):402-404.

63. Yu M, Takanishi D, Myers SA, Takiguchi SA, Severino R, Hasaniya N, Levy MM, McNamara JJ: **Frequency of mortality and myocardial infarction during maximizing oxygen delivery: a prospective, randomized trial.** *Crit Care Med* 1995, **23**(6):1025-1032.

64. Yu M, Pei K, Moran S, Edwards KD, Domingo S, Steinemann S, Ghows M, Takiguchi S, Tan A, Lurie F, Takanishi D,Jr: **A prospective randomized trial using blood volume analysis in addition to pulmonary artery catheter, compared with pulmonary artery catheter alone, to guide shock resuscitation in critically ill surgical patients.** *Shock* 2011, **35**(3):220-228.

65. Zhang JC, Ren HS, Jiang JJ, Ding M, Meng M, Zeng J, Chu YF, Zhu WY, Qi GQ, Wang P, Wang CT: **The effects of joint administration of 6% hydroxyethyl starch 130/0.4 and high-volume hemofiltration on patients with acute lung injury and acute kidney injury.** *Zhongguo Wei Zhong Bing Ji Jiu Yi Xue* 2011, **23**(12):755-758.
